# Supplementary material for: Emotional overeating affected nine in ten female students during the COVID-19 university closure: A cross-sectional study in France
Source: PLoS One. 2023 Aug 31;18(8):e0286439. doi: 10.1371/journal.pone.0286439 (PMC10470954; doi:10.1371/journal.pone.0286439)
Supplement: Attachment — Submitted filename: Response_to_reviewers_FINAL.docx [file pone.0286439.s001.docx]

**#1 Response to the editor**

Journal requirements:

When submitting your revision, we need you to address these additional requirements.

1. Please ensure that your manuscript meets PLOS ONE's style requirements, including those for file naming. The PLOS ONE style templates can be found at

<https://journals.plos.org/plosone/s/file?id=wjVg/PLOSOne_formatting_sample_main_body.pdf> and

<https://journals.plos.org/plosone/s/file?id=ba62/PLOSOne_formatting_sample_title_authors_affiliations.pdf>

ANSWER: Heading levels were corrected according to the submission guidelines, as well as table’s title. Manuscript’s text was double-spaced. A short title (<100 characters) was added. The names of the files were modified to fit with the standards.

2. We noticed you have some minor occurrence of overlapping text with the following previous publication(s), which needs to be addressed:

- <https://hal.science/hal-01856971v1/document>

- <https://doi.org/10.3389/fnut.2022.920170>

- <https://doi.org/10.1186/s12889-021-10531-3>

In your revision ensure you cite all your sources (including your own works), and quote or rephrase any duplicated text outside the methods section. Further consideration is dependent on these concerns being addressed.

ANSWER: These three references were added in the manuscript and rephrasing was made in some instances, except for the methods where the appropriate quotation was made without deeply modifying the text. The first two references were cited on the M&M section while the third one was added in the introduction, where evoking the higher prevalence of emotional eating in women compared to men.

3. "Thank you for stating the following financial disclosure:

“The present research was funded by the University of Rennes 1, Fondation de l’Avenir, the Benjamin Delessert Institute, and INRAE. A. Godet received a PhD grant from the University of Rennes 1.”

Please state what role the funders took in the study.  If the funders had no role, please state: "The funders had no role in study design, data collection and analysis, decision to publish, or preparation of the manuscript."

If this statement is not correct you must amend it as needed.

Please include this amended Role of Funder statement in your cover letter; we will change the online submission form on your behalf."

ANSWER: The following statement was added in the funding section and in the cover letter: “The funders had no role in study design, data collection and analysis, decision to publish, or preparation of the manuscript.”

4. We note that you have stated that you will provide repository information for your data at acceptance. Should your manuscript be accepted for publication, we will hold it until you provide the relevant accession numbers or DOIs necessary to access your data. If you wish to make changes to your Data Availability statement, please describe these changes in your cover letter and we will update your Data Availability statement to reflect the information you provide.

ANSWER: The data are already available and we provided the corresponding weblink in the “Data Availability Statement” section (p.26): “The datasets generated and analyzed for this study are available at the following address: <https://doi.org/10.57745/87KZFH>”

5. Please review your reference list to ensure that it is complete and correct. If you have cited papers that have been retracted, please include the rationale for doing so in the manuscript text, or remove these references and replace them with relevant current references. Any changes to the reference list should be mentioned in the rebuttal letter that accompanies your revised manuscript. If you need to cite a retracted article, indicate the article’s retracted status in the References list and also include a citation and full reference for the retraction notice.

ANSWER: Two studies (Brooks et al. 2020 and Topp et al., 2015) were corrected in the required citing style, and three other studies were added in the reference list: Coquery et al. (2022), McCullagh, P., & Nelder, J.A (1989) and Sze et al. (2021).

**2. Review Comments to the Author**

**Reviewer #1**: INTRODUCTION: in line 74 where it is stated: “Thirdly, women have genetic predisposition for higher impulsivity and higher reward sensitivity, which are associated with dopamine dysregulation during comfort eating”.

I suggest substantiating the assertion, using more studies supporting it, or in any case affirm that this was observed in “one” particular study, instead of generalizing.

ANSWER: This assumption was based on a review article; we accordingly added this precision, thank you for the clarification.

DISCUSSION: in line 308 it is affirmed: “Finally, DIO seemed more frequent in smokers and less frequent in younger students, which may correlate with indicators of academic stress”

I suggest reviewing this affirmation, because the linear regression analysis shows a negative association between DIO and age

ANSWER: Thank you for highlighted this mistake, the sentence was corrected accordingly “Finally, DIO seemed more frequent in smokers, which may correlate with indicators of academic stress (Nichter et al. 2007), and less frequent in older students.” (p.19)

**Reviewer #2**: I suggest the following aspects be completed or appear in the article:
It does not appear as a criterion for non-inclusion of participants, if they having a diagnosis of depression or antidepressant treatment or sleeping pills.

ANSWER: Thank you for your comment. This precision was added in the manuscript lines [105-106] “Being aged less than 18 and more than 24 years old were the only non-inclusion criteria. Any medical information was excluded from data collection.”

It isn't clear whether physical activity and hours of sleep were evaluated as a control variable, it is important because they could be different in each participant and may affect the study variables.

ANSWER: These variables were not controlled in the present study. We added this statement in the limits section lines [326]. “Third, some behavioural factors related to emotional overeating (*e.g.* sleep and physical activity) were not assessed”

The results do not present the R2 of the regression models, it is important to include the data to know the fit of the model.

ANSWER: The R2 are not indicated nor interpreted in the generalized linear models (GLM). A sentence and reference have been added accordingly in the M&M: “The pseudo R-squared is not discussed in generalized linear models texts (McCullagh & Nelder, 1989).”

The limitations of the study are recognized, but the inaccuracy of the weight and height measurements to calculate the BMI must be recognized, this could be under or over estimated, also if the control variables of physical activity, diagnosis of depression or treatment were not considered with antidepressants or sleeping pills, you should clarify it in the limitations.

ANSWER: We added this precision following the limitations of the lack of reported physical activity and sleeping information, line [327] “, and BMI estimates could have been biased due to self-reported data.”

**Reviewer #3**: The objective of the present investigation was to estimate the proportion of college women who reported overeating in response to emotions during the COVID-19 closure of universities, and to investigate the social and psychological factors associated with this stress response. This topic is extremely important, firstly because there are few studies that deal with the psychological state and secondly because this population already tends to have various affectations at this level.
To obtain their data, they applied an online questionnaire, which is a good method to obtain data remotely in an isolation situation, such as the COVID-19 pandemic.
The authors use adequate and validated tools for the population studied.
The University of Rennes has an enrollment of approximately 30,000 students, the authors recruited 300 students, this is one of the limitations of the study.
This study is important as it confirms that isolation due to the COVID-19 pandemic had a negative impact at different levels, especially in the psychological aspect. That 9 out of 10 students reported emotional overeating behaviors is alarming. This study will help to design interventions aimed at improving the psychological state of students, since many of the disorders adopted during the isolation due to the pandemic are still maintained today

ANSWER: Thank you for your comment. Even though we reckon that the potential of recruitment at the University of Rennes 1 is around 30,000 male and female students, it is important to precise that our recruitment campaign was performed in only one campus of the University (Beaulieu) and that only female volunteers aged 18-24yo who expressed an interest in this study were recruited. Previous descriptive studies have been performed and published with a similar number of volunteers (*e.g.* Constant et al. 2018).
